# Temporal trends in *Salmonella* serovar distribution and antimicrobial resistance profiles, including plasmid-mediated colistin resistance, among livestock-derived isolates in South Korea, 2019–2024

**DOI:** 10.3389/fmicb.2026.1821233

**Published:** 2026-05-22

**Authors:** Md. Sekendar Ali, Hee-Seung Kang, Bo-Youn Moon, Ji-In Kim, Yu-Jeong Hwang, Ji-Hyun Choi, Yeon-Hee Lee, Ha-Young Kim, Jae-Myung Kim, Suk-Kyung Lim

**Affiliations:** Bacterial Disease Division, Animal and Plant Quarantine Agency, Ministry of Agriculture, Food and Rural Affairs, Gimcheon, Republic of Korea

**Keywords:** IncX4 and IncI2 plasmids, livestock, *mcr-1*, multidrug resistance, *S*. Infantis

## Abstract

Amid the global threat of emerging antimicrobial resistance, the transmission of multidrug-resistant (MDR) *Salmonella* from livestock remains a critical concern for public health. This study investigated the longitudinal trends of *Salmonella* serovars and the molecular epidemiology of colistin resistance in 4,198 isolates from cattle, pigs, chickens, and ducks in South Korea (2019–2024). Among the 93 serovars identified, *S*. Infantis (40.1% in chickens), *S*. Typhimurium and variant (49.8% in pigs), and *S*. Typhimurium (39.1% in ducks) were predominant, with *S*. Infantis showing a dramatic surge in poultry during 2022–2024. Overall, 78.7% of isolates were resistant to at least one antimicrobial, and 50.5% were MDR; *S*. Infantis exhibited a particularly high MDR rate (90.7%), including significant resistance to the high-priority critically important antimicrobial cefotaxime (85.3%). Molecular characterization of colistin-resistant isolates revealed the presence of the mobile colistin resistance gene, *mcr-1*, localized on highly transmissible IncX4 and IncI2 plasmids in *S*. Infantis and *S*. Typhimurium, respectively. Comparative genomics via next-generation sequencing (NGS) showed over 99% homology between these plasmids and previously reported human clinical plasmids, and their horizontal transferability was confirmed through conjugation assays. These findings highlight a critical epidemiological shift toward hyper-resistant *S*. Infantis and the persistence of mobile *mcr-1* within the livestock reservoir, posing a substantial risk of zoonotic transmission through the food chain. This study underscores the urgent need for enhanced global surveillance and stricter antimicrobial stewardship to mitigate the dissemination of last-resort resistance at the animal-human interface.

## 1 Introduction

*Salmonella* remains a paramount global public health challenge, acting as a leading cause of bacterial foodborne gastroenteritis worldwide. It is estimated to be responsible for approximately 93.8 million cases of human invasive infections annually (Galán-Relaño et al., 2023). Over the past decade, major salmonellosis outbreaks linked to contaminated food have been reported worldwide, including in the USA, Thailand, and China (Pornsukarom et al., 2015; Hoffmann et al., 2020; Wang et al., 2025). In South Korea, the epidemiological burden is equally significant; *Salmonella* accounts for 3.5% of all water- and foodborne disease outbreaks nationwide, affecting over 55,700 individuals each year (Park et al., 2015).

While non-typhoidal salmonellosis often presents as self-limiting, severe infections necessitate effective antimicrobial therapy, particularly in vulnerable populations such as children, the elderly, and immunocompromised patients (Ranjan et al., 2026). Food-producing animals, including chickens, cattle, pigs, and ducks, serve as the primary reservoirs for *Salmonella* transmission to humans via the food chain (Ferrari et al., 2019). Consequently, monitoring the distribution and temporal trends of *Salmonella* serovars in livestock is a critical pillar of public health surveillance (Skrzypiec et al., 2025). Such data are essential for identifying emerging dominant serovars, evaluating the efficacy of farm-level biosecurity measures, and implementing targeted food safety interventions (Huang, 2024).

Furthermore, as specific serovars are often associated with distinct antimicrobial resistance (AMR) profiles, longitudinal monitoring is vital for informing risk assessments and clinical management practices. The escalating emergence of multidrug-resistant (MDR) *Salmonella* in the livestock sector, driven by the extensive use of antimicrobials for prophylaxis and therapy, has become a global crisis (Gut et al., 2018). While beta-lactams and fluoroquinolones are the primary clinical choices for treating MDR infections, the rising resistance to these “critically important” antimicrobials severely limits therapeutic options (Enshaie et al., 2025). Of even greater concern is the emergence of resistance to “last-resort” antibiotics, such as colistin, often mediated by mobile genetic elements, such as the *mcr* genes, which facilitate rapid horizontal transmission across bacterial species (Touati et al., 2025).

In South Korea, despite ongoing efforts through the Korean Veterinary Antimicrobial Resistance Monitoring System (KVARS), the dynamic shifts in *Salmonella* serovars and their evolving resistance landscapes—particularly in emerging lineages like *S*. Infantis—require urgent and updated investigation. Therefore, the objective of this study was to analyze the trends in serovar distribution and antimicrobial resistance profiles of *Salmonella* spp. isolated from feces and carcasses of livestock in South Korea between 2019 and 2024. Furthermore, we performed high-resolution molecular characterization of colistin-resistant *Salmonella* isolates to elucidate the genetic mechanisms and plasmid-mediated transmissibility of the *mcr-1* gene.

## 2 Materials and methods

### *2.1 Salmonella* isolation and identification

A total of 4,198 *Salmonella* isolates were obtained from cattle (*n* = 55), pigs (*n* = 580), chickens (*n* = 2,604), and ducks (*n* = 959). The strains were collected from 16 laboratories/centers involved in the Korean Veterinary Antimicrobial Resistance Monitoring System (KVARS) from 2019 to 2024. Isolation, identification, and serovaring of *Salmonella* from cattle, pig, chicken, and duck carcasses and feces were conducted using the previously described methodology (Mechesso et al., 2020). The isolation procedure for *Salmonella* involves pre-enriching the sample in buffered peptone water and incubating it in a modified semisolid Rappaport-Vassiliadis medium (Becton Dickinson, CA, USA). The colonies were grown on CHROMagar (Merck, Darmstadt, Germany) and identified using matrix-assisted laser desorption ionization-time-of-flight mass spectrometry (Bruker Corporation, MA, USA). The *Salmonella* serovars were then detected using a total of 10 specific primers by polymerase chain reaction (PCR). The list of primers and reaction conditions was described in Supplementary Table S1. We performed slide agglutination targeting the *Salmonella* O and H antigens by the White-Kauffman-Le Minor technique for other serovars (Grimont and Weill, 2007).

### 2.2 Antimicrobial susceptibility assessment

The 4,198 *Salmonella* isolates were tested for antimicrobial susceptibility using the broth microdilution method with the Sensititre^TM^ panel KRNVF (Thermo Fisher, Waltham, USA) to determine the minimum inhibitory concentrations (MICs) of the tested antimicrobials. *E. coli* ATCC (American Type Culture Collection) 25922 served as a quality reference strain. The determined MICs were evaluated in accordance with the Clinical and Laboratory Standards Institute (CLSI) 2025 (CLSI, 2025) and the National Antimicrobial Resistance Monitoring System (NARMS) 2023 (NARMS, 2023). Colistin susceptibility was assessed by MIC testing for all *Salmonella* spp., except serovar D, which showed intrinsic resistance. The bacterial broth culture was made turbid equivalent to a 0.5 McFarland standard, followed by incubation for 16–20 h at 35 ± 2 °C. We have determined the colistin resistance based on the breakpoint ≥4 μg/mL (CLSI, 2025). Antimicrobial resistance and MDR were excluded for colistin. MDR was defined as the isolates that were resistant to at least one agent from each of three classes of antimicrobials (Magiorakos et al., 2012).

### 2.3 Characterization of colistin-resistant *Salmonella* spp.

We performed polymerase chain reaction (PCR) to detect the *mcr* genes in two colistin-resistant isolates (*S*. Infantis 20S2003 and *S*. Typhimurium 23E02001). The PCR conditions and primers were described previously (Rebelo et al., 2018).

The conjugation test was performed using the filter-mating technique with rifampin-resistant *E. coli* RG488 as the recipient strain and *S*. Infantis 20S2003 and *S*. Typhimurium 23E02001 as donors (Ali et al., 2024). The donor and recipient strains were cultured overnight, followed by incubation in tryptic soy broth (Becton Dickinson, CA, USA) and subsequently incubated for 4 h at 37 °C. The newly cultured bacteria were mated at a donor-to-recipient ratio (1:4), followed by retaining them on a membrane filter. The transconjugants were isolated by inoculating the mixture onto MacConkey agar plates supplemented with rifampin (50 μg/mL) and ceftiofur (25 μg/mL). The selected transconjugants were assessed for antimicrobial susceptibility based on the CLSI guidelines (CLSI, 2025). Moreover, the presence of *mcr-1* in the transconjugant was also confirmed by PCR.

### 2.4 Next-generation sequencing (NGS) analysis

Colistin-resistant *Salmonella* isolates were subjected to NGS using the Illumina MiSeq (Illumina Inc., CA, USA) and Oxford Nanopore (Nanopore Technologies Ltd., Oxford, UK) platforms. Genomic DNA was extracted and purified using the tissue kit (Qiagen, Hilden, Germany). Genomic libraries were constructed for short-read sequencing utilizing the Illumina DNA Prep (M) Tagmentation kit (Illumina Inc., CA, USA), and sequencing was performed on the Illumina MiSeq platform by outsourcing to Macrogen (Macrogen Inc., Seoul, South Korea). The long-read libraries were prepared in-house using the Ligation Sequencing Kit V14 (SQK-LSK114) (Nanopore Technologies Ltd., Oxford, UK). Sequencing was accomplished in-house using MinION, followed by basecalling with Dorado (version 1.3.1) to obtain the full genome sequence. We conducted quality control on the Illumina and Nanopore sequencing data and subsequently performed assembly using Unicycler (version 0.4.8) (Wick et al., 2017). Genome annotation was performed by Prokka v1.14.6 (Seemann, 2014). Antimicrobial resistance (AMR) genes were identified using ResFinder (version 2.1) along with the Comprehensive Antibiotic Resistance Database (CARD), while virulence factors were assessed using the Virulence Factor Database (VFDB) (version 5.0). Furthermore, mobile genetic elements, visualization, and characteristics of circular genome maps were examined using Proksee (https://proksee.ca/).

### 2.5 Statistical analysis

We used Microsoft Excel 2020 (Microsoft Corporation, Redmond, USA) and Rex Software (version 3.0.3, RexSoft, Inc., Seoul, South Korea) to analyze the data. The chi-square test was employed to determine differences in antimicrobial resistance rates between groups, with *p*-values < 0.05 considered statistically significant.

## 3 Results

### 3.1 *Salmonella* serovars

In total, 93 *Salmonella* serovars were found in food animals: 14 in cattle, 42 in pigs, 61 in chickens, and 44 in ducks (Tables 1–4). The distribution of serovars was different based on animal species: in cattle, *S*. Agona (20%) and *S*. Rissen (18.2%) were predominantly detected, while in pigs, *S*. Typhimurium variant (26.7%) and *S*. Typhimurium (23.1%); in chickens, *S*. Infantis (40.1%) and *S*. Enteritidis (9.9%); and in ducks, *S*. Typhimurium (39.1%) and *S*. Enteritidis (12.8%) were prevalent. The two most predominant serovars in cattle, pigs, chickens, and ducks accounted for over 50% of the total serovars isolated from their respective animals.

**Table 1:** Distribution of *Salmonella* serovars isolated from cattle during 2019–2024 in South Korea.

Serovars	% (no. of isolates)		Subtotal	*p*-value^*^
	2019–2021	2022–2024		
*S*. Agona	12.0 (3)	26.7 (8)	20.0 (11)	0.17
*S*. Rissen	36.0 (9)	3.3 (1)	18.2 (10)	< 0.01
*S*. Infantis	8.0 (2)	10.0 (3)	9.1 (5)	0.79
*S*. Bareilly	16.0 (4)	0 (0)	7.3 (4)	0.02
*S*. Newport	0 (0)	13.3 (4)	7.3 (4)	0.05
*S*. Typhimurium	0 (0)	10.0 (3)	5.5 (3)	0.10
*S*. Enteritidis	4.0 (1)	6.7 (2)	5.5 (3)	0.66
*S*. Typhimurium variant	12.0 (3)	0 (0)	5.5 (3)	0.05
*S*. Senftenberg	0 (0)	10.0 (3)	5.5 (3)	0.10
*S*. Albany	8.0 (2)	0 (0)	3.6 (2)	0.11
*S*. Derby	4.0 (1)	3.3 (1)	3.6 (2)	0.89
Others^**^	0 (0)	16.7 (5)	9.1 (5)	0.03
Total	25	30	55	

^*^p < 0.05 indicates significant changes in the trend of serovar distribution.

^**^3 serovars.

**Table 2:** Distribution of *Salmonella* serovars isolated from pigs during 2019–2024 in South Korea.

Serovars	% (no. of isolates)		Subtotal	*p*-value^*^
	2019–2021	2022–2024		
*S*. Typhimurium variant	27.6 (112)	24.6 (43)	26.7 (155)	0.45
*S*. Typhimurium	22.9 (93)	23.4 (41)	23.1 (134)	0.89
*S*. Rissen	15.0 (61)	4.0 (7)	11.7 (68)	<0.01
*S*. Derby	5.2 (21)	11.4 (20)	7.1 (41)	<0.01
*S*. Infantis	4.4 (18)	9.1 (16)	5.9 (34)	0.02
*S*. Agona	7.1 (29)	1.1 (2)	5.3 (31)	<0.01
*S*. Bareilly	2.0 (8)	1.1 (2)	1.7 (10)	0.48
*S*. Montevideo	1.5 (6)	1.1 (2)	1.4 (8)	0.75
*S*. Senftenberg	1.0 (4)	1.7 (3)	1.2 (7)	0.45
*S*. Duesseldorf	0.7 (3)	0 (0)	0.5 (3)	0.25
*S*. Enteritidis	0.7 (3)	0 (0)	0.5 (3)	0.25
*S*. Bousso	0.7 (3)	0 (0)	0.5 (3)	0.25
*S*. Anatum	0.7 (3)	0 (0)	0.5 (3)	0.25
Others^**^	10.3 (42)	21.8 (38)	13.8 (80)	<0.01
Total	406	174	580	

^*^p < 0.05 indicates significant changes in the trend of serovar distribution.

^**^29 serovars.

**Table 3:** Distribution of *Salmonella* serovars isolated from chickens during 2019–2024 in South Korea.

Serovars	% (no. of isolates)		Subtotal	*p*-value^*^
	2019–2021	2022–2024		
*S*. Infantis	9.3 (100)	61.8 (943)	40.1 (1043)	<0.01
*S*. Enteritidis	12 (129)	8.4 (128)	9.9 (257)	<0.01
*S*. Agona	14.9 (161)	3.9 (60)	8.5 (221)	<0.01
*S*. Montevideo	9.9 (107)	4.8 (73)	6.9 (180)	<0.01
*S*. Albany	12.1 (131)	0.1 (1)	5.1 (132)	<0.01
*S*. Senftenberg	3.1 (33)	5.4 (83)	4.5 (116)	<0.01
*S*. Westhampton	7.2 (78)	0.4 (6)	3.2 (84)	<0.01
*S*. Bareilly	4.2 (45)	0.5 (8)	3.1 (81)	<0.01
*S*. Typhimurium variant	3.9 (42)	1.6 (25)	2.6 (67)	<0.01
*S*. Virchow	4.4 (48)	0.2 (3)	2.0 (51)	<0.01
Others^**^	19.0 (205)	12.8 (195)	14.3 (372)	<0.01
Total	1,079	1,525	2,604	

^*^p < 0.05 indicates significant changes in the trend of serovar distribution.

^**^52 serovars.

**Table 4:** Distribution of *Salmonella* serovars isolated from ducks during 2019–2024 in South Korea.

Serovars	% (no. of isolates)		Subtotal	*p*-value^*^
	2019–2021	2022–2024		
*S*. Typhimurium	38.5 (196)	39.8 (179)	39.1 (375)	0.68
*S*. Enteritidis	18.3 (93)	6.7 (30)	12.8 (123)	<0.01
*S*. Albany	11.8 (60)	4.2 (19)	8.2 (79)	<0.01
*S*. Hadar	1.4 (7)	15.6 (70)	8.0 (77)	<0.01
*S*. Typhimurium variant	9.4 (48)	3.6 (16)	6.7 (64)	<0.01
*S*. Indiana	3.3 (17)	4.7 (21)	4.0 (38)	0.29
*S*. Uganda	0 (0)	7.3 (33)	3.4 (33)	<0.01
*S*. London	3.5 (18)	2.9 (13)	3.2 (31)	0.57
*S*. Give	2.0 (10)	3.1 (14)	2.5 (24)	0.25
*S*. Infantis	0.2 (1)	2.4 (11)	1.3 (12)	<0.01
Others^**^	11.6 (59)	9.8 (44)	10.7 (103)	0.36
Total	509	450	959	

^*^p < 0.05 indicates significant changes in the trend of serovar distribution.

^**^34 serovars.

There were changes in the distribution of serovars across all livestock species between 2019–2021 and 2022–2024. The most predominant serovar remained unchanged, except for chickens. However, in cattle, *S*. Rissen (36% vs. 3.3%), *S*. Bareilly (16% vs. 0%), and *S*. Typhimurium variants (12% vs. 0%) decreased in 2022–2024 compared to 2019–2021, but *S*. Newport (0% vs. 13.3%) increased in 2022–2024 (*p* < 0.05). In pigs, *S*. Rissen (15% vs. 4%) and *S*. Agona (7.1% vs. 1.1%) decreased, *S*. Derby (5.2% vs. 11.4%) and *S*. Infantis (4.4% vs. 9.1%) increased (*p* < 0.05). In chickens, the prevalence of all the top 10 serovars was changed. In note, *S*. Infantis (9.3% vs. 61.8%) dramatically increased while the other 9 serovars decreased, except *S*. Senftenberg (3.1% vs. 5.4%). In ducks, the prevalence of *S*. Hadar (1.4% vs. 15.6%), *S*. Uganda (0% vs. 7.3%), and *S*. Infantis (0.2% vs. 2.4%) was increased, while *S*. Enteritidis (18.3% vs. 6.7%), *S*. Albany (11.8% vs. 4.2%), and *S*. Typhimurium variants (9.4% vs. 3.6%) decreased (*p* < 0.05). Overall, the prevalence of *S*. Typhimurium and *S*. Typhimurium variants was stably distributed in pigs and ducks; however, *S*. Infantis was increased in all livestock except cattle.

### 3.2 Antimicrobial resistance

Of the tested antimicrobials, resistance to nalidixic acid in *Salmonella* isolates was the highest (62.9%), followed by ampicillin (48.5%), sulfisoxazole (47.4%), tetracycline (45.1%), and streptomycin (43%). However, amoxicillin/clavulanic acid, cefepime, cefoxitin, ceftazidime, and ciprofloxacin resistance rates were low (< 5%). At the animal species level, the antimicrobial resistance rates of isolates obtained from cattle, pigs, and chickens were generally similar, although they were comparatively lower in duck isolates (Table 5). In pig and cattle isolates, ampicillin resistance was the most prevalent (59.2% and 32.7%), followed by sulfisoxazole (57.1% and 54.5%), streptomycin (52.8% and 54.5%), and tetracycline (50.8% and 45.5%). In chickens, nalidixic acid resistance was the most frequent (74.7%), followed by ampicillin (54.8%), tetracycline (54.2%), sulfisoxazole (53.6%), and streptomycin (47.9%). Similarly, resistance to nalidixic acid was the maximum (54.5%), followed by ampicillin (26.1%), sulfisoxazole (24.2%), and tetracycline (16.8%) in duck isolates. In note, resistance to critically important antimicrobials, such as cefotaxime and gentamicin, was much higher in chickens (39.4% and 28.5%, respectively) than in other animal species (cattle, 12.7% and 16.4%; pigs, 20% and 13.1%, and ducks, 1% and 0.8%, respectively).

**Table 5:** Antimicrobial resistance in *Salmonella* isolated from livestock during 2019–2024 in South Korea.

Antimicro-bials	% (no. of resistant isolates)																			
	Cattle				Pigs				Chickens				Ducks				Total			
	2019–2021 (*n* = 25)	2022–2024 (*n* = 30)	Subtotal (*n* = 55)	*p*-value	2019–2021 (*n* = 406)	2022–2024 (*n* = 174)	Subtotal (*n* = 580)	*p*-value	2019–2021 (*n* = 1,079)	2022–2024 (*n* = 1,525)	Subtotal (*n* = 2,604)	*p*-value	2019–2021 (*n* = 509)	2022–2024 (*n* = 450)	Subtotal (*n* = 959)	*p*-value	2019–2021 (*n* = 2,019)	2022–2024 (*n* = 2,179)	Total (*n* = 4198)	*p*-value
AMC	8.0 (2)	0 (0)	3.6 (2)	0.11	1.7 (7)	0 (0)	1.2 (7)	0.08	1.8 (19)	0 (0)	0.7 (19)	<0.01	0 (0)	0.2 (1)	0.1 (1)	0.28	1.4 (28)	0 (1)	0.7 (29)	<0.01
AMP	32.0 (8)	33.3 (10)	32.7 (18)	0.91	56.9 (231)	64.6 (113)	59.2 (344)	0.08	43.9 (474)	62.5 (952)	54.8 (1,426)	<0.01	32.0 (163)	19.3 (87)	26.1 (250)	<0.01	43.4 (876)	53.4 (1,162)	48.5 (2,038)	<0.01
FEP	4.0 (1)	0 (0)	1.8 (1)	0.26	1.7 (7)	10.9 (19)	4.5 (26)	<0.01	1.6 (17)	1.6 (25)	1.6 (42)	0.90	0.2 (1)	0 (0)	0.1 (1)	0.34	1.3 (26)	2.0 (44)	1.7 (70)	0.06
CTX	20.0 (5)	6.7 (2)	12.7 (7)	0.13	6.4 (26)	20.0 (35)	10.5 (61)	<0.01	10.6 (115)	59.8 (911)	39.4 (1,026)	<0.01	0.4 (2)	1.8 (8)	1.0 (10)	0.03	7.3 (148)	43.9 (956)	26.3 (1,104)	<0.01
FOX	8.0 (2)	0 (0)	3.6 (2)	0.11	3.7 (15)	1.7 (3)	3.1 (18)	0.20	1.6 (17)	2.8 (43)	2.3 (60)	0.04	0.4 (2)	0.2 (1)	0.3 (3)	0.63	1.8 (36)	2.1 (47)	2.0 (83)	0.44
CAZ	12.0 (3)	0 (0)	5.5 (3)	0.05	1.7 (7)	9.1 (16)	4.0 (23)	<0.01	4.1 (44)	1.8 (27)	2.7 (71)	<0.01	0.2 (1)	0 (0)	0.1 (1)	0.34	2.7 (55)	2.0 (43)	2.3 (98)	0.10
CHL	16.0 (4)	53.3 (16)	36.4 (20)	<0.01	30.5 (124)	50.3 (88)	36.5 (212)	<0.01	30.1 (325)	58.3 (888)	46.6 (1,213)	<0.01	9.2 (47)	10.2 (46)	9.7 (93)	0.60	24.8 (500)	47.7 (1,038)	36.6 (1,538)	<0.01
CIP	0 (0)	0 (0)	0 (0)	ND	4.7 (19)	2.3 (4)	4.0 (23)	0.17	3.7 (40)	1.8 (27)	2.6 (67)	<0.01	4.7 (24)	7.8 (35)	6.2 (59)	0.04	4.1 (83)	3.0 (66)	3.5 (149)	0.05
GEN	24.0 (6)	10.0 (3)	16.4 (9)	0.16	17.0 (69)	13.1 (23)	15.8 (92)	0.24	9.4 (101)	42.2 (642)	28.5 (743)	<0.01	0.2 (1)	1.6 (7)	0.8 (8)	0.02	8.8 (177)	31.0 (675)	20.3 (852)	<0.01
MEM	0 (0)	0 (0)	0 (0)	ND	0 (0)	0 (0)	0 (0)	ND	0 (0)	0 (0)	0 (0)	ND	0 (0)	0 (0)	0 (0)	ND	0 (0)	0 (0)	0 (0)	ND
NAL	56.0 (14)	16.7 (5)	34.5 (19)	<0.01	28.8 (117)	21.1 (37)	26.5 (154)	0.05	63.2 (683)	82.9 (1,262)	74.7 (1,945)	<0.01	54.4 (277)	54.7 (246)	54.5 (523)	0.93	54.0 (1,091)	71.2 (1,550)	62.9 (2,641)	<0.01
STR	32.0 (8)	73.3 (22)	54.5 (30)	<0.01	47.0 (191)	66.3 (116)	52.8 (307)	<0.01	30.3 (327)	60.5 (921)	47.9 (1,248)	<0.01	24.2 (123)	22.0 (99)	23.1 (222)	0.42	32.1 (649)	53.2 (1,158)	43.0 (1,807)	<0.01
FIS	36.0 (9)	70.0 (21)	54.5 (30)	0.01	53.0 (215)	66.9 (117)	57.1 (332)	<0.01	42.3 (457)	61.7 (939)	53.6 (1,396)	<0.01	34.0 (173)	13.1 (59)	24.2 (232)	<0.01	42.3 (854)	52.2 (1,136)	47.4 (1,990)	<0.01
TET	28.0 (7)	60.0 (18)	45.5 (25)	0.01	49.0 (199)	54.9 (96)	50.8 (295)	<0.01	40.0 (432)	64.3 (980)	54.2 (1,412)	<0.01	20.2 (103)	12.9 (58)	16.8 (161)	<0.01	36.7 (741)	52.9 (1,152)	45.1 (1,893)	<0.01
SXT	20.0 (5)	13.3 (4)	16.4 (9)	0.50	15.5 (63)	22.9 (40)	17.7 (103)	0.03	31.3 (338)	38.1 (580)	35.3 (918)	<0.01	14.5 (74)	7.8 (35)	11.4 (109)	0.07	23.8 (480)	30.3 (659)	27.1 (1,139)	<0.01
MDR	40.0 (10)	63.3 (19)	52.7 (29)	0.08	59.6 (242)	74.7 (130)	64.1 (372)	<0.01	44.9 (484)	65.2 (994)	56.8 (1,478)	<0.01	30.6 (156)	18.7 (84)	25.0 (240)	<0.01	44.2 (892)	56.3 (1,226)	50.5 (2,118)	<0.01

AMC, amoxicillin/clavulanic acid; AMP, ampicillin; CAZ, ceftazidime; CHL, chloramphenicol; CIP, ciprofloxacin; CTX, cefotaxime; FEP, cefepime; FIS, sulfisoxazole; FOX, cefoxitin; GEN, gentamicin; MEM, meropenem; NAL, nalidixic acid; STR, streptomycin; SXT, trimethoprim/sulfamethoxazole; TET, tetracycline. MDR, multidrug resistance. ND, not defined. *p* < 0.05 indicates significant changes in the trend of antimicrobial resistance.

We found the change in antimicrobial resistance between 2019–2021 and 2022–2024. The prevalence of resistant isolates in cattle, pigs, and chickens was greater in 2022–2024 than in 2019–2021, except for ducks (Table 5). Overall, trends of antimicrobial resistance varied by animal species and antimicrobials. Chloramphenicol, streptomycin, sulfisoxazole, and tetracycline resistance increased in cattle (16% vs. 53.3%, 32% vs. 73.3%, 36% vs. 70%, and 28% vs. 60%, respectively), pigs (30.5% vs. 50.3%, 47% vs. 66.3%, 53% vs. 66.9%, 49% vs. 54.9%, respectively), and chickens (30.1% vs. 58.3%, 30.3% vs. 60.5%, 42.3% vs. 61.7%, 40% vs. 64.3%) in 2022–2024 than in 2019–2021; however, amoxicillin/clavulanic acid (8% vs. 0%, 1.7% vs. 0%, 1.8% vs 0%, respectively) and cefoxitin (20% vs. 6.7%, 6.4% vs. 20%, 10.6% vs. 59.8%, respectively) resistance decreased in 2022–2024 (*p* < 0.05). Notably, resistance to cefotaxime dramatically increased in pigs (6.4% vs. 20%) and chickens (10.6% vs. 59.8%) in 2022–2024 (*p* < 0.05). Moreover, resistance to ciprofloxacin decreased in all animals (cattle, 0% vs. 0%; pigs, 4.7% vs. 2.3%, and chickens, 3.7% vs. 1.8%, respectively) except ducks (4.7% vs. 7.8%).

The antimicrobial resistance rates of the 10 most frequent *Salmonella* isolates against the tested antimicrobials are shown in Table 6. Overall, resistance rates for the most prevalent serovars were greater than those of less common serovars. Antimicrobial resistance rates (>80%) to ampicillin, cefotaxime, chloramphenicol, nalidixic acid, streptomycin, tetracycline, and sulfisoxazole were predominantly detected in *S*. Infantis. Similarly, higher nalidixic acid (100%), sulfisoxazole (100%), and trimethoprim/sulfamethoxazole (100%), ampicillin (72.4%), chloramphenicol (74.8%), and tetracycline (71%) resistance was found in *S*. Albany isolates. In *S*. Agona, resistance rates (>50%) to ampicillin, chloramphenicol, streptomycin, tetracycline, and sulfisoxazole were also highly identified. *S*. Typhimurium and *S*. Typhimurium variants also showed a high resistance rate to ampicillin, streptomycin, and sulfonamide. Furthermore, the overall resistance in the *S*. Typhimurium variant was higher than that of *S*. Typhimurium.

**Table 6:** Antimicrobial resistance in the most frequent *Salmonella* isolated from livestock during 2019–2024 in South Korea.

Antimicro-bials	% (no. of resistant isolates)											
	*S*. Infantis (*n* = 1094)	*S*. Typhimurium (*n* = 553)	*S*. Enteritidis (*n* = 386)	*S*. Typhimurium variant (*n* = 289)	*S*. Agona (*n* = 268)	*S*. Albany (*n* = 214)	*S*. Montevideo (*n* = 195)	*S*. Senftenberg (*n* = 132)	*S*. Bareilly (*n* = 114)	*S*. Rissen (*n* = 92)	Others^*^(*n* = 861)	Total (*n* = 4,198)
AMC	0 (0)	0.2 (1)	0 (0)	2.1 (6)	0.4 (1)	1.4 (3)	0 (0)	0 (0)	1.8 (2)	2.2 (2)	1.6 (14)	0.7 (29)
AMP	87.6 (958)	29.7 (164)	54.4 (210)	63.7 (184)	58.2 (156)	72.4 (155)	2.1 (4)	5.3 (7)	6.1 (7)	32.6 (30)	18.9 (163)	48.5 (2,038)
FEP	0.4 (4)	0.5 (3)	8.8 (34)	5.2 (15)	0 (0)	0 (0)	0.5 (1)	0 (0)	0 (0)	0 (0)	1.5 (13)	1.7 (70)
CTX	85.3 (933)	2.9 (16)	12.4 (48)	11.1 (32)	3.7 (10)	1.4 (3)	1.0 (2)	0 (0)	4.4 (5)	4.3 (4)	5.9 (51)	26.3 (1,104)
FOX	3.8 (42)	1.1 (6)	0.3 (1)	3.8 (11)	1.1 (3)	1.9 (4)	0 (0)	0 (0)	1.8 (2)	2.2 (2)	1.4 (12)	2.0 (83)
CAZ	0.4 (4)	0.7 (4)	10.4 (40)	6.2 (18)	1.1 (3)	0.9 (2)	0.5 (1)	0 (0)	1.8 (2)	2.2 (2)	2.6 (22)	2.3 (98)
CHL	83.8 (917)	17.4 (96)	2.6 (10)	23.5 (68)	55.6 (149)	74.8 (160)	1.0 (2)	5.3 (7)	5.3 (6)	26.1 (24)	11.5 (99)	36.6 (1,538)
CIP	0.8 (9)	3.1 (17)	1.3 (5)	1.0 (3)	4.1 (11)	15.4 (33)	5.1 (10)	1.5 (2)	0 (0)	3.3 (3)	6.5 (56)	3.5 (149)
GEN	61.4 (672)	9.8 (54)	11.4 (44)	6.9 (20)	1.1 (3)	0.9 (2)	2.1 (4)	0 (0)	6.1 (7)	13.0 (12)	3.9 (34)	20.3 (852)
MEM	0 (0)	0 (0)	0 (0)	0 (0)	0 (0)	0 (0)	0 (0)	0 (0)	0 (0)	0 (0)	0 (0)	0 (0)
NAL	91.2 (998)	39.2 (217)	95.6 (369)	22.5 (65)	15.7 (42)	100 (214)	90.8 (177)	82.6 (109)	8.8 (10)	41.3 (38)	46.7 (402)	62.9 (2,641)
STR	83.5 (914)	19.3 (107)	47.7 (184)	50.5 (146)	65.3 (175)	11.7 (25)	5.1 (10)	7.6 (10)	6.1 (7)	30.4 (28)	23.3 (201)	43.0 (1,807)
FIS	86.3 (944)	20.8 (115)	47.2 (182)	49.5 (143)	67.9 (182)	100 (214)	6.2 (12)	6.8 (9)	3.5 (4)	28.3 (26)	18.5 (159)	47.4 (1,990)
TET	90.0 (985)	17.4 (96)	35.2 (136)	44.3 (128)	67.5 (181)	71.0 (152)	5.6 (11)	6.8 (9)	4.4 (5)	43.5 (40)	17.4 (150)	45.1 (1,893)
SXT	56.9 (622)	9.6 (53)	1.8 (7)	14.2 (41)	47.4 (127)	100 (214)	4.1 (8)	0.8 (1)	2.6 (3)	16.3 (15)	5.6 (48)	27.1 (1,139)
MDR	90.7 (992)	23.9 (132)	54.7 (211)	57.4 (166)	67.5 (181)	81.3 (174)	7.2 (14)	7.6 (10)	6.1 (7)	39.1 (36)	22.8 (196)	50.5 (2,118)

AMC, amoxicillin/clavulanic acid; AMP, ampicillin; CAZ, ceftazidime; CHL, chloramphenicol; CIP, ciprofloxacin; CTX, cefotaxime; FEP, cefepime; FIS, sulfisoxazole; FOX, cefoxitin; GEN, gentamicin; MEM, meropenem; NAL, nalidixic acid; STR, streptomycin; SXT, trimethoprim/sulfamethoxazole; TET, tetracycline. MDR, multidrug resistance.

^*^83 serovars.

Additionally, the highest overall resistance was observed for nalidixic acid, with the serovars *S*. Enteritidis, *S*. Montevideo, and *S*. Senftenberg all demonstrating resistance levels exceeding 80% (Table 6). The *S*. Enteritidis showed a broader resistance profile. In addition to high nalidixic acid resistance, it exhibited significant resistance (over 30%) to several other antibiotics, including ampicillin, streptomycin, sulfisoxazole, and tetracycline. In contrast, the *S*. Montevideo and *S*. Senftenberg displayed low resistance to most agents. Apart from their high resistance to nalidixic acid, all other tested antimicrobials showed resistance rates below 10% for both *S*. Montevideo and *S*. Senftenberg.

Resistance to high-priority critically important antimicrobials (HP-CIA) was generally low but showed specific spikes: cefotaxime resistance was notably high in the *S*. Infantis, reaching 85.3% (Table 6). Other serovars, such as *S*. Enteritidis and *S*. Typhimurium variant, showed much lower resistance, at approximately the 10% level. Resistance to other HP-CIA antimicrobials was below 5% across the board. Resistance to ciprofloxacin was highest in *S*. Albany (15.4%). Other serovars exhibited lower rates, including *S*. Montevideo at 5.1% and *S*. Albany, *S*. Typhimurium, and *S*. Rissen at 3–4%. The “Other” group also registered a 6.5% resistance rate, with the main contribution coming from *S*. Kentucky. Resistance to colistin was rare, confirmed in only one strain each from the *S*. Infantis and *S*. Typhimurium. Colistin resistance was observed in 0.05% (2/3778) in the non-*Salmonella* D group.

### 3.3 Multidrug resistance (MDR) and antimicrobial resistance patterns

The MDR and antimicrobial resistance patterns of the major serovars are presented in Tables 5–7 and Supplementary Tables S2–S11. We found that 78.7% of the isolates in this investigation showed resistance to one or more antimicrobial agents. Moreover, more than half of the *Salmonella* isolates were MDR, with the highest detection in pigs (64.1%), followed by chickens (56.8%), cattle (52.7%), and ducks (25%) (Table 5). In addition, the MDR rate was >40% in five predominant serovars, with *S*. Infantis (90.7%) exhibiting the maximum prevalence, followed by *S*. Albany (81.3%), *S*. Agona (67.5%), *S*. Typhimurium variant (57.4%), and *S*. Enteritidis (54.7%) (Table 6).

**Table 7:** Antimicrobial resistance pattern in *Salmonella* (n = 4,198) isolated from livestock during 2019–2024 in South Korea.

Number of antimicrobials	% (No. of resistant isolates)	Most common pattern (No. of isolates)
0	21.3 (895)	–
1	20.7 (867)	NAL (n = 713)
2	6.4 (270)	NAL FIS (n = 76)
3	3.8 (158)	NAL FIS SXT (n = 43)
4	6.6 (277)	AMP NAL STR FIS (n = 79)
5	5.9 (247)	AMP NAL STR FIS TET (n = 81)
6	9.3 (392)	AMP CHL STR FIS TET SXT (n = 139)
7	5.1 (215)	AMP CTX CHL NAL STR FIS TET (n = 62)
8	9.4 (394)	AMP CTX CHL GEN NAL STR FIS TET (n = 191)
9	10.7 (450)	AMP CTX CHL GEN NAL STR FIS TET SXT (n = 411)
10	0.7 (29)	AMP CTX FOX CHL GEN NAL STR FIS TET SXT (n = 19)
11	0.1 (3)	AMC AMP CTX FOX CAZ CHL NAL STR FIS TET SXT (n = 1),
		AMP CTX FOX CHL CIP GEN NAL STR FIS TET SXT (n = 1),
		AMP CTX FOX CAZ CHL GEN NAL STR FIS TET SXT (n = 1)

AMP, ampicillin; CAZ, ceftazidime; CHL, chloramphenicol; CTX, cefotaxime; FEP, cefepime; FIS, sulfisoxazole; GEN, gentamicin; NAL, nalidixic acid; STR, streptomycin; SXT, trimethoprim/sulfamethoxazole; TET, tetracycline.

The most common pattern of resistance to a single antimicrobial was nalidixic acid (17%) resistance (Table 7). This resistance pattern was predominantly found in *S*. Senftenberg (80.3%), *S*. Montevideo (72.3%), and *S*. Enteritidis (37%) (Supplementary Tables S4, S8–S9). Similarly, resistance to ampicillin, cefotaxime, chloramphenicol, gentamicin, nalidixic acid, streptomycin, tetracycline, trimethoprim/sulfamethoxazole, and sulfisoxazole was highly detected in *S*. Infantis (35.4%) (Supplementary Table S2). In addition, the resistance patterns that include five or more antimicrobial agents were predominantly found among *S*. Albany (66.8%), *S*. Infantis (63%), *S*. Agona (47.8%), *S*. Enteritidis isolates (28.5%), and *S*. Typhimurium variant (13.5%) (Supplementary Tables S2, S4–S7).

### 3.4 Molecular characterization of colistin-resistant *Salmonella*

The *mcr-1* gene was detected in two isolates, *S*. Infantis and *S*. Typhimurium, from pig carcasses and pig diarrheal feces, respectively. Furthermore, the resistance pattern of *S*. Infantis includes resistance to colistin, and *S*. Typhimurium includes ampicillin, chloramphenicol, gentamicin, nalidixic acid, streptomycin, and trimethoprim/sulfamethoxazole resistance (Table 8). The conjugation assay revealed that *S*. Infantis showed transferability of the colistin resistance, and *S*. Typhimurium demonstrated colistin, chloramphenicol, streptomycin, and trimethoprim/sulfamethoxazole resistance transfer to the recipient *E. coli* RG488 (Table 8).

**Table 8:** Characterization of *mcr-1*-carrying *Salmonella* isolated from livestock during 2019–2024 in South Korea.

Isolates	Animals	Samples	Year	Province	Serovars	ST	Resistance pattern	Transferred resistance
20S02003	Pig	Carcass	2020	Gyeongbuk	*S*. Infantis	32	COL	COL
23E02001	Pig	Diarrheal feces	2021	Jeju	*S*. Typhimurium	19	AMP, CHL, COL, GEN, NAL, STR, SXT	AMP, CHL, COL, STR, SXT

AMP, ampicillin; CHL, chloramphenicol; COL, colistin; GEN, gentamicin; NAL, nalidixic acid; STR, streptomycin; SXT, trimethoprim/sulfamethoxazole. ST, sequence type.

**Figure 1:**
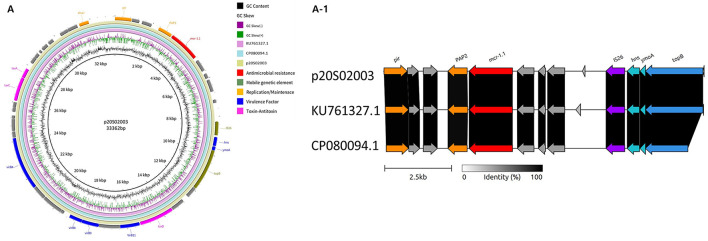
Structural and comparative analysis of plasmid p20S02003. **(A)** Circular plasmid (IncX4) maps of *mcr-1.1*-carrying *S*. Infantis 20S02003 and two reference strains (*K. pneumoniae* SZ04 KU761327.1 and *S*. Typhimurium variant SLR1_8245 CP080094.1). Different genes and plasmid structural features are colored by their functions. **(A-1)** Comparison of the genetic context of *S*. Infantis 20S02003 with *K. pneumoniae* SZ04 KU761327.1 and *S*. Typhimurium variant SLR1_8245 CP080094.1 in their linear structures. Arrows indicate gene position and transcriptional direction. Conserved genes, including *pir, PAP2*, insertion sequence IS*26*, and neighboring genes *(hns, ymoA, topB)* are shown. Shaded regions represent nucleotide sequence similarity (0–100%). Scale bar, 2.5 kb.

The IncX4 plasmid, approximately 33 kbp in size, analyzed in this study from an *S*. Infantis isolate (20S2003), contained the *mcr-1* gene, which was positioned adjacent to *pap2* (Figure 1 and Table 9). Circular map analysis confirmed the typical structure, featuring an IS*26* insertion sequence surrounding the *mcr-1* gene. Comparative genomics revealed that this plasmid exhibited 99% homology with previously reported IncX4 plasmids: pMCR1-IncX4 from a clinical *K. pneumoniae* SZ04 isolate (GenBank: KU761327.1) and an IncX4 plasmid from a monophasic *S*. Typhimurium variant SLR1_8245 (GenBank: CP080094.1). Crucially, the arrangement of the taxABC cluster, pilX operon, replication, and transfer regions was completely conserved, demonstrating structural uniformity despite the difference in bacterial species, *K. pneumoniae* and *Salmonella*.

**Table 9:** Molecular characteristics of the colistin-resistant *Salmonella* isolated from livestock during 2019–2024 in South Korea.

Isolate	Source	Year	*mcr*	Phenotype	Serovar	Resistance gene location	AMR gene	p. Mutation	Base pair (bp)	Virulence factor
20S2003	Pig carcass	2020	*mcr-1*	COL	*S*. Infantis	Chromosome	*aac(6′)-Iaa*	*parC* p.T57S	4,616,741	*mgtC, mgtB, misL, lpfA, lpfB, lpfC, lpfD, lpfE, sopD, invH, invF, invG, invE, invA, invB, invC, invI, invJ, spaO, spaP, spaQ, spaR, spaS, sicA, sipB/sspB, sipC/sspC, sipD, sipA/sspA, sicP, sptP, prgH, prgI, prgJ, prgK, orgA, orgB, orgC, avrA, mig-14, pipB2, sinH, ratB, shdA, sseL, sspH2, sseK2, sopA, sopE2, steC, sseJ, steB, sifB, steA, ssaU, ssaT, ssaS, ssaR, ssaQ, ssaP, ssaO, ssaN, ssaV, ssaM, ssaL, ssaK, ssaJ, ssaI, ssaH, ssaG, sseG, sseF, sscB, sseE, sseD, sseC, sscA, sseB, sseA, ssaE, ssaD, ssaC, spiC/ssaB, sifA, csgC, csgA, csgB, csgD, csgE, csgF, csgG, sopB/sigD, pipB, sopD2, slrP, fimF, fimH, fimD, fimC, fimI, sseK1*
						Plasmid 1 [IncX4]	*mcr-1.1*	–	33,362	–
23E02001	Pig diarrheal feces	2021	*mcr-1*	AMP CHL COL GEN NAL STR SXT FIS	*S*. Typhimurium	Chromosome	*aac(6′)-Iaa*	*gyrA* p.D87Y	4,814,120	*SPI-1(invA, invB, invC, invE, invF, invG, invH, invI, invJ; spaO, spaP, spaQ, spaR, spaS; sipA/sspA, sipB/sspB, sipC/sspC, sipD; sicA, sicP; sptP; prgH, prgI, prgJ, prgK; orgA, orgB, orgC), SPI-2(ssaC, ssaD, ssaE, ssaG, ssaH, ssaI, ssaJ, ssaK, ssaL, ssaM, ssaN, ssaO, ssaP, ssaQ, ssaR, ssaS, ssaT, ssaU, ssaV; sseA, sseB, sseC, sseD, sseE, sseF, sseG, sseJ, sseI/srfH, sseK1, sseK2, sseL; sscA, sscB; spiC/ssaB; sifA, sifB; pipB, pipB2; steA, steB, steC; slrP; gogB), SPI-3(mgtB, mgtC, misL), SPI-5(sopB/sigD, sopD, sopE2), Independent effectors(avrA, sopA, sopD2, mig-14, sinH, sspH2, grvA), Adhesion and colonization (lpfA, lpfB, lpfC, lpfD, lpfE; ratB, shdA), Curli/fimbriae (fimC, fimD, fimF, fimH, fimI; csgA, csgB, csgC, csgD, csgE, csgF, csgG), Stress resistance (sodCI)*
						Plasmid 1 [IncFIB]	*aph(4)-Ia, aadA1, aadA2, aac(3)-IVa*, *bla*_TEM − 1_, *cml1, qacL, sul3, tet(M), dfrA12*	–	135,658	–
						Plasmid 2 [IncI1]	*aph(6)-Id, aph3(^′′^)-Ib*		85,289	
						Plasmid 3 [IncI2]	*mcr-1.1*		60,960	

AMP, ampicillin; CHL, chloramphenicol; COL, colistin; GEN, gentamicin; NAL, nalidixic acid; STR, streptomycin; SXT, trimethoprim/sulfamethoxazole; FIS, sulfisoxazole. AMR, antimicrobial resistance.

**Figure 2:**
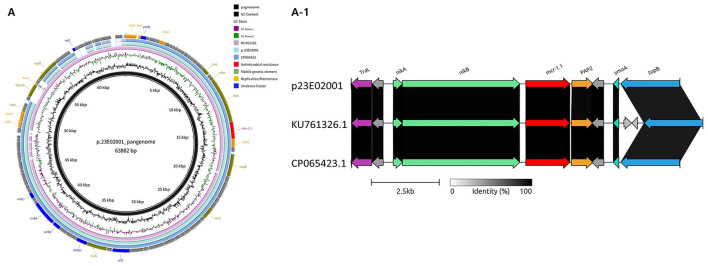
Structural and comparative analysis of plasmid p23E02001. **(A)** Circular plasmid (IncI2) maps of *mcr-1.1*-carrying *S*. Typhimurium 23E02001 and two reference strains (*E. coli* SZ02 KU761326 and *S*. Typhimurium K18JST013 CP065423.1). Different genes and plasmid structural features are colored by their functions. **(A-1)** Comparison of the genetic context of *S*. Typhimurium 23E02001 with *E. coli* SZ02 KU761326 and *S*. Typhimurium K18JST013 CP065423.1 in their linear structures. Arrows indicate gene position and transcriptional direction. Conserved genes, including *nikA, nikB, PAP2, ymoA*, and *topB* are shown. Shaded regions represent nucleotide sequence similarity (0–100%). Scale bar, 2.5 kb.

Plasmid replicon analysis identified an IncI2 plasmid (approx. 65 kb) in *S*. Typhimurium (23E02001) carrying the *mcr-1* gene (Figure 2 and Table 9). Whole-genome sequencing and circular map alignment revealed an extremely high homology (99.92%) with the Korean *S*. Typhimurium plasmid pK18JST013 (CP065423.1). This similarity spanned the entire backbone, including the repA-tra/pil region and the arrangement of the mcr-1-pap2 module adjacent to nikB. A notable common feature was the absence of prominent flanking insertion sequences (IS) adjacent to the mcr-1-pap2 cassette in both the current isolate and CP065423.1.While general similarity to the international reference plasmid pMCR1-IncI2 (*E. coli* SZ02 KU761326) was maintained, the current plasmid showed closer identity to the Korean isolate. Additionally, this isolate co-harbored IncFIB (approx. 135.6 kb) and IncI1 (approx. 85.3 kb) plasmids.

Other resistance genes, including those for beta-lactams (*bla*_TEM − 1_), aminoglycosides (*AAC(3)-IVa*, APH family), trimethoprim/sulfamethoxazole (*dfrA12/sul3*), tetracycline (*tet*(M)), and disinfectants (*qacL*), were segregated and distributed primarily across the IncFIB and IncI1 plasmids (Table 9). In addition, point mutation in the chromosomal DNA, resulting in amino acid substitutions at T57S in *parC* in *S*. Infantis and D87Y in *gyrA* in *S*. Typhimurium, which are linked to quinolone resistance.

Screening for virulence factors using the VFDB database demonstrated that a number of different virulence factors are present in *S*. Infantis and *S*. Typhimurium (Table 9). The identified virulence genes encompassed those responsible for *Salmonella* pathogenicity island*: invA, invB, invC, invE, invF, invG, invH, invI, invJ;* independent effectors *(avrA, sopA, sopD2, mig-14, sinH, sspH2, grvA);* adhesion and colonization: *lpfA, lpfB, lpfC, lpfD, lpfE;* fimbriae: *fimC, fimD, fimF, fimH, fimI;* and stress resistance*: sodCI*.

## 4 Discussion

This study characterizes temporal trends in *Salmonella* serovar distribution and antimicrobial resistance profiles, including colistin resistance mediated by plasmid-borne *mcr-1*, among livestock-derived isolates in South Korea from 2019 to 2024. Crucially, we also identified that the *mcr-1* gene is carried on highly transmissible IncX4 and IncI2 plasmids with over 99% homology to human clinical isolates, suggesting the potential risk for last-resort resistance dissemination from livestock through the food chain.

Diverse clinically significant *Salmonella* serovars have been detected in food animals in our study. Similar to our investigation, *S*. Infantis has been identified as the most common serovar in Korean chickens (Kang et al., 2024a). Similarly, this serovar was predominantly identified in food animals globally, including in Slovenia (Papić et al., 2022) and Chile (Lapierre et al., 2020). The portion of *S*. Enteritidis among the duck isolates was lower than in a previous study (40.8%) (Yang et al., 2019). However, it was consistent with the findings of (Park et al. 2015) in Korea (11.8%) regarding ducks.

Prior research has consistently found *S*. Agona from cattle in Korea, while *S*. Rissen was less frequent (Song et al., 2024). The prevalence of *S*. Agona has increased among food animals worldwide. This is alarming given the recent outbreaks of *S*. Agona in humans reported in China (Wang et al., 2025), the USA (Hoffmann et al., 2020), and Korea (Lee et al., 2021). The percentage of *S*. Rissen (18.2%) among cattle isolates was higher than reported in our previous study (Mechesso et al., 2020). Moreover, it was lower in Italy (38%) (Bolzoni et al., 2025). *S*. Rissen is also among the most prevalent serovars in humans and food animals, responsible for outbreaks of foodborne disease, especially in Asia (Pornsukarom et al., 2015).

The prevalence of *S*. Typhimurium monophasic variant (*S*. 1,4,[5],12:i:-) has significantly increased worldwide, including in Korea. In this investigation, the proportion of *S*. 1,4,[5],12:i:- among the pig isolates was greater than an earlier report in Korea (14.5%) (Mechesso et al., 2020). It has been identified as one of the most prevalent serovars among pigs in the European Union (EU) (Campos et al., 2019). Moreover, in China (Qin et al., 2022), this serovar was also frequently detected in pigs. *S*. 1,4,[5],12:i:- has been identified in the food chain, encompassing the food animals and their products (Sun et al., 2020). Since *S*. 1,4,[5],12:i:- is one of the common causes of human salmonellosis outbreaks, the increase in its proportion in pigs is concerning.

Prior research has consistently detected *S*. Infantis and *S*. Enteritidis serovars in chickens in Korea (Kim et al., 2012; Kang et al., 2024a,b), although *S*. Agona was comparatively low (Lee et al., 2021). Furthermore, over the past few years, the prevalence of *S*. Infantis in chickens has increased dramatically (Kang et al., 2024a). Additionally, despite inconsistencies, the proportion of *S*. Agona was elevated in poultry. This is worrying because recent human outbreaks of *S*. Agona have been reported (Hoffmann et al., 2020; Wang et al., 2025).

*Salmonellosis* caused by ingestion of infected poultry products is a recognized public health issue. The prevalence of serovar *S*. Enteritidis in chickens in our investigation aligned with other findings (Zakaria et al., 2022) but was lower than the proportions documented by Kim et al. (2012) (57.4%). This serovar is a major contributor to *Salmonella*-associated foodborne outbreaks worldwide (Luo et al., 2021). Although the overall proportion of *S*. Albany among chicken isolates was lower than in our previously published reports in Korea (90.1%) (Ali et al., 2023), this serovar remains a concern for human salmonellosis.

*S*. Typhimurium was found as one of the prevalent serovars in ducks in Korea (20.5%) (Park et al., 2023); similarly, we have detected a high occurrence of this serovar (39.1%) among duck isolates. The increased prevalence of *S*. Typhimurium may lead to human salmonellosis through the consumption of meat from food animals or through direct contact. The proportion of *S*. Enteritidis identified in duck isolates in this investigation was somewhat more than in prior research (8.8%) (Cho et al., 2011), posing a concern for human health.

Variations in the proportions and distributions of serovars may be attributed to spatial and temporal disparities across studies. Moreover, differences might be due to slaughterhouse management facilities, animal age, sample type, sampling season, and isolation procedures (Lee et al., 2016). A total of 13 commonly isolated serovars among cattle, pigs, chickens, and ducks in this study, which are also listed in the World Health Organization's top 20 serovars associated with human salmonellosis globally (Hendriksen et al., 2011). Of them, *S*. Albany, *S*. Agona, *S*. Typhimurium, *S*. Infantis, *S*. Enteritidis, *S*. Virchow, and *S*. Rissen were recurrently reported in humans in Asian countries, particularly in Korea (Tamang et al., 2011; Lee et al., 2021). The infrequently recovered serovars *S*. Derby, *S*. London, and others in food animals in this investigation were also described in human patients in Korea (Kim et al., 2022). These findings suggest the possibility that different *Salmonella* serovars in food-producing animals can be transmitted to humans through direct contact or the food chain. Therefore, initiatives to mitigate human salmonellosis attributed to these serovars should prioritize the implementation of control measures in food animals.

The prevalence of antimicrobial-resistant *Salmonella* is a major global public health issue. Differences in antimicrobial resistance rates were detected among serovars. Concurring with previous reports in Korea (Ali et al., 2023, 2024), a greater proportion of *Salmonella* isolates from cattle, pigs, chickens, and ducks exhibited resistance to ampicillin, streptomycin, sulfisoxazole, and tetracycline. *S*. Infantis*, S*. Albany, *S*. Agona, *S*. Enteritidis, *S*. Typhimurium, and *S*. 1,4,[5],12:i:- were the most common serovars showing resistance to these antimicrobials. Likewise, earlier research conducted in Chile (Lapierre et al., 2020) and Korea (Tamang et al., 2015; Kang et al., 2024b) showed that human and food animal *S*. Infantis, *S*. Albany, *S*. Agona, *S*. Enteritidis, *S*. Typhimurium, and *S*. 1,4,[5],12:i:- had greater rates of resistance to ampicillin, streptomycin, sulfisoxazole, and tetracycline. The significant resistance to these antimicrobials can be explained by the fact that aminoglycosides, β-lactams, and tetracyclines are generally used in Korean livestock for the treatment and management of bacterial infections (APQA, 2022). The persistent application of these antimicrobials in the veterinary sector may impose selection pressure on *Salmonella*, thereby promoting the emergence of resistant strains.

Despite the prohibition of chloramphenicol in food animals in Korea (Song et al., 2022), 36.6% of *Salmonella* serovars, including significant proportions of *S*. Infantis (83.8%), *S*. Albany (74.8%), and *S*. Agona (55.6%), exhibited resistance to this antimicrobial. This might be partially attributed to the ongoing consumption of alternative phenicols, including florfenicol in food animals (Song et al., 2024). Additionally, the use of antimicrobials such as tetracycline and trimethoprim/sulfamethoxazole may promote the emergence of *Salmonella* that exhibit resistance to other unrelated antimicrobials, including chloramphenicol, attributable to the genetic connection of resistance genes (Acar, 2015).

The development of clinically important antimicrobial resistance in *Salmonella*, including cephalosporin and fluoroquinolone, poses a global public health issue. Our investigation revealed that an overall prevalence of cefotaxime resistance at 26.3%, with the highest resistance observed in *S*. Infantis (85.3%), followed by *S*. Enteritidis (12.4%) and *S*. 1,4,[5],12:i:- (11.1%). The resistance in *S*. Infantis is consistent with the reports in Chile (64.4%) (Lapierre et al., 2020) and Italy (20.7%) (Proietti et al., 2020). However, resistance in *S*. Enteritidis and *S*. 1,4,[5],12:i:- was much higher than that previously described in China (2.5%) (Yang et al., 2023). Resistance to cefotaxime in *Salmonella* serovars, including *S*. Infantis and *S*. Enteritidis, has been reported in food animals and humans in Korea (Tamang et al., 2015; Ali et al., 2025). The structural and resistance mechanisms resemblance among third-generation cephalosporins may lead to the development of strains resistant to other clinically important cephalosporins, including ceftiofur.

Consistent with previous investigations in Korea (Kim et al., 2014), about 60% *Salmonella* isolates in this study exhibited resistance to nalidixic acid. Nonetheless, this was lower than that described in China (39.2%) (Kuang et al., 2015). The serovars *S*. Infantis, *S*. Enteritidis, *S*. Albany, *S*. Montevideo, and *S*. Senftenberg exhibited the highest resistance rate to nalidixic acid. The extensive use of enrofloxacin in food-producing animals, particularly chickens in Korea, may be contributing to the increased prevalence of resistance to nalidixic acid. Moreover, resistance to nalidixic acid may result in the development of strains resistant to fluoroquinolones, including ciprofloxacin. Ciprofloxacin resistance was detected in *S*. Albany (15.4%) and other serovars, including *S*. Montevideo, *S*. Agona, *S*. Typhimurium, and *S*. Rissen at 3–5.1%. Notwithstanding certain inconsistencies in resistance relative to findings in Korea and China (Li et al., 2017; Mechesso et al., 2020; Ali et al., 2025), the current study revealed that these serovars exhibited an increase in resistance to this antimicrobial frequently used in both humans and food animals. Ciprofloxacin resistance rate in this study aligns with that reported by (Mechesso et al. 2020) in Korea, but differs from the findings by (Li et al. 2017) in China. Furthermore, this investigation corroborates reports of increased incidence of ciprofloxacin-resistant *S*. Albany and *S*. Typhimurium in Thailand (Sinwat et al., 2016). These findings are alarming as ciprofloxacin is one of the most critically important antimicrobials recommended for treating *Salmonella* infections in humans (Tamang et al., 2015).

Colistin is considered one of the last-resort antimicrobials used for the treatment of multidrug-resistant bacterial infections. However, the emergence of colistin-resistant *Salmonella* in humans and livestock complicates the treatment options. Colistin resistance was found in *S*. Infantis (0.1%) and *S*. Typhimurium (0.2%). Consistent with this study, earlier research conducted in Korea (Kang et al., 2024a) and other countries (Haoran et al., 2021; Zakaria et al., 2022) has also detected a low prevalence of *Salmonella* (0.1–1.1%) resistance to colistin in chickens, pigs, and cattle. Conversely, a comparatively higher occurrence of colistin-resistant *Salmonella* was isolated from livestock in Egypt (15.8%) (El Baz et al., 2025).

Approximately 80% of *Salmonella* isolates from cattle, pigs, chickens, and ducks demonstrated resistance to at least one antimicrobial, which is consistent with a previous investigation (Yang et al., 2010). These food animals serve as reservoirs of *Salmonella*, and the use of antimicrobials for treatment and disease prevention hastens the emergence of resistance. The overall prevalence of MDR *Salmonella* in this investigation (50.5%) was concordant with findings in Vietnam (44%) (Thai et al., 2012); however, it was lower than in other studies, including in China (72.3%) (Biao et al., 2022). MDR was prevalent in *S*. Infantis, *S*. Albany, *S*. Agona, *S*. Enteritidis, and *S*. 1,4,[5],12:i:-, aligning with prior reports conducted in Asia (Phongaran et al., 2019; Mechesso et al., 2020).

In accordance with Yang et al. (2010), resistance to nalidixic acid was the primary resistance pattern observed for a single antimicrobial among major serovars, including *S*. Enteritidis, *S*. Montevideo, and *S*. Senftenberg. Moreover, the majority of serovars, particularly *S*. Albany, *S*. Infantis, *S*. Agona, *S*. 1,4,[5],12:i:-, and *S*. Enteritidis, include five or more antimicrobial resistance patterns, including resistance to ampicillin, cefotaxime, chloramphenicol, gentamicin, nalidixic acid, streptomycin, tetracycline, trimethoprim/sulfamethoxazole, and sulfisoxazole. In addition, resistance to these antimicrobials in the serovars has been observed to increase recently. Likewise, *Salmonella* isolates from food animals in Korea demonstrated a similar resistance pattern (Kang et al., 2024a). Furthermore, consistent with published studies carried out in Italy (Proietti et al., 2020) and the USA (Hoffmann et al., 2020), resistance to these antimicrobials was normally observed in *S*. Infantis, *S*. Infantis, and *S*. Agona isolates from livestock. Moreover, in alignment with the previous research, which found that *S*. 1,4,[5],12:i:- and *S*. Enteritidis from food-producing animals commonly demonstrated resistance to ampicillin, chloramphenicol, gentamicin, nalidixic acid, streptomycin, tetracycline, and sulfisoxazole (Piryaei et al., 2025).

In this study, we found that *S*. Infantis and *S*. Typhimurium, isolated from pig carcasses and diarrheal feces, respectively, carried the *mcr-1* gene. The food-producing animals were often found to harbor plasmid-mediated resistance genes, such as *mcr-1*, which are associated with the development of colistin resistance in *Salmonella*. Furthermore, *Salmonella* carrying the *mcr-1* gene has often been identified in Korean livestock (Moon et al., 2021). Thus, the emergence of resistance to this antimicrobial in *Salmonella* isolates from food animals requires vigilant monitoring.

The conjugation assay demonstrated that both of the *mcr-1*-carrying *S*. Infantis and *S*. Typhimurium isolates exhibited the transferability of resistance to the recipient *E. coli* RG488. The hybrid assembly results explicitly localized the *mcr-1.1* gene to a ~33.4-kb IncX4 plasmid in *S*. Infantis and a ~61.0-kb IncI2 plasmid in *S*. Typhimurium. Consistent with this finding, a prior study found that *Salmonella* isolated from food animals could transfer the *mcr-1* gene to a recipient via conjugation (Anyanwu et al., 2021). Furthermore, the conjugation analysis revealed a successful horizontal transfer of colistin resistance; the colistin MIC increased from ≤ 0.5 μg/mL in the recipient *E. coli* RG488 to ≥8 μg/mL in the transconjugants. Moreover, our findings suggested that the *mcr-1* gene was co-transferred with chloramphenicol, streptomycin, and trimethoprim/sulfamethoxazole resistance, corroborating earlier research demonstrating that *S*. Typhimurium isolates also co-transferred these antimicrobial resistances with the colistin resistance gene (Haoran et al., 2021). Additionally, the horizontal spread of *mcr-1* and other genes, such as *AmpR* (ampicillin resistance gene), *catA* (chloramphenicol resistance gene), *aac6*′*)-Ie-aph2*′'*)-Ia* (aminoglycoside resistance gene), and *strA* (streptomycin resistance) in *S*. Typhimurium was investigated previously (Gonçalves, 2023). The result of our current investigation indicates that elevated resistance to these antimicrobials is potentially associated with the *mcr-1*-harboring plasmid in *S*. Typhimurium.

The *S*. Infantis IncX4 plasmid identified here shares the identical structure with existing IncX4-type mcr-1 plasmids. The mcr-1-pap2-IS26 element constitutes a conserved mobile genetic unit, consistent with findings by Kuleshov et al. (2021), reinforcing the potential for stable, strain-independent *mcr-1* transmission. However, a key observation is that our *S*. Infantis isolate carried *mcr-1* without displaying a multidrug resistance (MDR) phenotype. While prior reports show *S*. Infantis is primarily disseminated globally with an MDR pattern often linked to pESI-like megaplasmids—where the combination of MDR and *mcr-1* presents a maximal public health threat—this isolate exclusively harbors *mcr-1* via the IncX4 plasmid, lacking the MDR background (Papić et al., 2022). This finding suggests that the spread of *mcr-1* can occur independently of the MDR.

We found that the IncI2 plasmid harboring *mcr-1* revealed a high degree of homology with the reference *S*. Typhimurium plasmid. Moreover, this similarity extended across the entire backbone, encompassing the repA-tra/pil region and the arrangement of the mcr-1-pap2 module inserted between nikB and topB. However, a significant shared characteristic was the lack of flanking IS next to the mcr-1-pap2 cassette in both the present isolate and the reference one. The present plasmid displayed a closer identity to the Korean isolate, suggesting a possible localized clustering or lineage. Additionally, the resistance genes, encompassing those for beta-lactams, aminoglycosides, trimethoprim/sulfamethoxazole, tetracycline, and disinfectants, were predominantly located on the IncFIB and IncI1 plasmids, contributing to their resistance (Moon et al., 2021).

The identification of multiple plasmids harboring *mcr-1* in a single *Salmonella* strain has significant evolutionary and pathogenicity implications. The co-presence of redundant *mcr-1* copies can augment the colistin resistance stability by reducing the likelihood of resistance loss through plasmid segregation (Alba et al., 2021). The existence of distinct plasmid backbones enhances the possibility of horizontal gene transfer, facilitating the spread of *mcr-1* among various pathogenic bacteria. Furthermore, multiple plasmids may increase the opportunity of recombination events, generating mosaic plasmids associating *mcr-1* with supplementary antimicrobial resistance determinants, thus accelerating the development of MDR (Portes et al., 2022). Therefore, this event can typically reflect the selective pressure of antimicrobials on *Salmonella* from food animals, potentially disseminating resistance to humans.

Fluoroquinolone resistance in *Salmonella* is often associated with mutations in the subunits of topoisomerase II (*gyrA*) and IV (*parC*) within the quinolone resistance-determining regions (QRDRs) (Ali et al., 2025). In this investigation, the mutations were identified in *parC* at T57S and in *gyrA* at D87Y. Resistance to nalidixic acid in *S*. Infantis generally possesses the T57S mutation in *parC* along with other mutations in QRDRs (Aslantaş and Türkyilmaz, 2025). Furthermore, the mutation at D87Y in *gyrA* of the QRDRs induces fluoroquinolone resistance in *S*. Typhimurium (Ali et al., 2025).

In this study, numerous virulence factors were identified. Among them, factors for adhesion and colonization (*lpf)* are essential for biofilm formation, exacerbating the antimicrobial resistance in *Salmonella* (Yang et al., 2025). The *Salmonella* pathogenicity island (SPI) genes facilitate colonization, invasion, and survival of this genus in the unfavorable environment (Sia et al., 2025). Similarly, genes encoding independent effectors (*avr)* facilitate the invasion of *Salmonella* into epithelial cells. In addition, the stress resistance gene (*sodCI)* contributes to survival and stimulates systemic dissemination of *Salmonella* in mammalian hosts (Fenlon, 2020).

## 5 Conclusion

This study demonstrates a significant shift in *Salmonella* serovar distribution and antimicrobial resistance profiles of *Salmonella* among South Korean livestock from 2019 to 2024, most notably the rapid emergence of MDR *S*. Infantis in poultry. The high resistance rates to clinically vital antimicrobials, including third-generation cephalosporins and quinolones, pose a direct challenge to public health and food safety. Of particular concern is the identification of the *mcr-1* gene on highly transmissible IncX4 and IncI2 plasmids in *S*. Infantis and *S*. Typhimurium. The structural conservation of these plasmids suggests a high potential for the horizontal dissemination of “last-resort” resistance through the food chain. Given the close serovar correlation between food animals and human clinical cases, our findings underscore the urgent need for integrated molecular investigation and more stringent antimicrobial stewardship and One Health surveillance to mitigate the zoonotic transmission of high-risk MDR *Salmonella* lineages.

### 5.1 Limitations and future perspective

It is worth mentioning that this study has some limitations. Differences in the numbers and distributions of *Salmonella* serovars may be due to longitudinal and spatial variations of the study. Furthermore, variations may arise from slaughterhouse and farm management systems, age of selected animals, sampling types and seasons, and isolation protocols. Especially, we cannot rule out potential cross-contamination of carcasses in the slaughterhouse, which should be carefully considered in future investigations.

## Data Availability

The original contributions presented in the study are included in the article/Supplementary material, further inquiries can be directed to the corresponding author/s.
