# Supplementary material for: Temporal trends in Salmonella serovar distribution and antimicrobial resistance profiles, including plasmid-mediated colistin resistance, among livestock-derived isolates in South Korea, 2019–2024
Source: Front Microbiol. 2026 May 22;17:1821233. doi: 10.3389/fmicb.2026.1821233 (PMC13236912; doi:10.3389/fmicb.2026.1821233)
Supplement: Supplementary file 1 [file Table_1.docx]

***Supplementary Materials***

Supplementary Table S1. List of *Salmonella* serovar polymerase chain reaction (PCR) primers

| Serovar | Primer name | Sequence (5’-3’) | Annealing Temp. (℃) | Amplicon Size (bp) | Reference |
| --- | --- | --- | --- | --- | --- |
| *S.* Typhimurium  /*S.* Typhimurium variant | mdh-F | TTC CAC CAC GCC CTT C | 55 | 505 | (Hong et al., 2023) |
|  | mdh-R | GCC GGG TAT GGA CCG TTC |  |  |  |
|  | fljb-hin-F | CGC TGG GTG ATT TCA GCC TGG AT | 55 | 1434 | (Hong et al., 2023) |
|  | fljb-hin-R | GAA TAT CGT CAC CGC TCT GCG ATT T |  |  |  |
| *S.* Enteritidis | ENT-F | TGT GTT TTA TCT GAT GCA AGA GG | 57 | 304 | (de Freitas et al., 2010) |
|  | ENT-R | TGA ACT ACG TTC GTT CTT CTG G |  |  |  |
| *S.* Agona | 33842-F | TAT CTA TTG GCT GGA TTG AC | 58 | 322 | (Ye et al., 2021a) |
|  | 33842-R | TCA TTC TCC TGA CCT ATA GT |  |  |  |
| *S.* Indiana | 36780-F | CCA ATG CTT TTG CCG TTC TA | 60 | 646 | (Ye et al., 2021a) |
|  | 36780-R | TTT CCC GAC GCA AGT TCA TA |  |  |  |
| *S.* Derby | 36441-F | CTG AAT GAA GCA GCA ATG GT | 62 | 457 | (Ye et al., 2021a) |
|  | 36441-R | GAG AAT CAG GGC GTT TCC AT |  |  |  |
| *S.* Infantis | 878-F | TTG CTT CAG CAG ATG CTA AG | 56 | 413 | (Bugarel et al., 2017) |
|  | 1275-R | CCA CCT GCG CCA ACG CT |  |  |  |
| *S.* Choleraesuis | FlinC-F | AAG GAA AAG ATC ATG GCA CAA | 55 | 963 | (Chiu et al., 2005) |
|  | FlinC-R | GAA CCC ACC ATC AAT AAC TTT G |  |  |  |
| *S.* Albany | 27289-F | GCG TTG AGG TTG AGT GGT TG | 60 | 1187 | (Ye et al., 2021b) |
|  | 27289-R | GAA CAG CAA ATC ACG GTA GT |  |  |  |
| *S.* Hadar | 22774-F | GGA ATA ACA AAG GTG GTA CT | 60 | 902 | (Ye et al., 2021b) |
|  | 22774-R | CCT GAC CTT AGA GAA TGG CT |  |  |  |

**References**

Bugarel, M., Tudor, A., Loneragan, G. H., and Nightingale, K. K. (2017). Molecular detection assay of five *Salmonella* serotypes of public interest: Typhimurium, Enteritidis, Newport, Heidelberg, and Hadar. *J. Microbiol. Methods* 134, 14–20. doi: 10.1016/j.mimet.2016.12.011

Chiu, T.-H., Pang, J.-C., Hwang, W.-Z., and Tsen, H.-Y. (2005). Development of PCR primers for the detection of *Salmonella enterica* serovar Choleraesuis based on the fliC gene. *J. Food Prot.* 68, 1575–1580. doi: 10.4315/0362-028X-68.8.1575

de Freitas, C. G., Santana, Â. P., da Silva, P. H. C., Gonçalves, V. S. P., Barros, M. de A. F., Torres, F. A. G., et al. (2010). PCR multiplex for detection of *Salmonella* Enteritidis, Typhi and Typhimurium and occurrence in poultry meat. *Int. J. Food Microbiol.* 139, 15–22. doi: 10.1016/j.ijfoodmicro.2010.02.007

Hong, Y., Ji, R., Wang, Z., Gu, J., Jiao, X., and Li, Q. (2023). Development and application of a multiplex PCR method to differentiate *Salmonella enterica* serovar Typhimurium from its monophasic variants in pig farms. *Food Microbiol.* 109, 104135. doi: 10.1016/j.fm.2022.104135

Ye, Q., Shang, Y., Chen, M., Pang, R., Li, F., Wang, C., et al. (2021a). Identification of new serovar-specific detection targets against *Salmonella* B serogroup using large-scale comparative genomics. *Food Control* 124, 107862. doi: 10.1016/j.foodcont.2020.107862

Ye, Q., Shang, Y., Chen, M., Pang, R., Li, F., Xiang, X., et al. (2021b). Identification of novel sensitive and reliable serovar-specific targets for PCR detection of *Salmonella* serovars Hadar and Albany by pan-genome analysis. *Front. Microbiol.* 12, 605984. doi: 10.3389/fmicb.2021.605984

Supplementary Table S2. Antimicrobial resistance pattern in *Salmonella* Infantis (n = 1,094) isolated from livestock during 2019–2024 in South Korea

| Number of antimicrobials | % (No. of resistant isolates) | Most common pattern (No. of isolates) |
| --- | --- | --- |
| 0 | 6.1 (67) | – |
| 1 | 2.7 (30) | NAL (n = 19) |
| 2 | 0.5 (5) | AMP CHL (n = 2) |
| 3 | 0.8 (9) | AMP CHL TET (n = 7) |
| 4 | 1.4 (15) | NAL STR FIS TET (n = 12) |
| 5 | 2.5 (27) | AMP CTX CHL NAL TET (n = 6) |
| 6 | 5.3 (58) | AMP CTX NAL STR FIS TET (n = 27) |
| 7 | 9.9 (108) | AMP CTX CHL NAL STR FIS TET (n = 60) |
| 8 | 32.6 (357) | AMP CTX CHL GEN NAL STR FIS TET (n = 190) |
| 9 | 36.3 (397) | AMP CTX CHL GEN NAL STR FIS TET SXT (n = 387) |
| 10 | 1.8 (20) | AMP CTX FOX CHL GEN NAL STR FIS TET SXT (n = 18) |
| 11 | 0.1 (1) | AMP CTX FOX CAZ CHL GEN NAL STR FIS TET SXT (n = 1) |

AMP, ampicillin; CAZ, ceftazidime; CHL, chloramphenicol; CTX, cefotaxime; FEP, cefepime; FIS, sulfisoxazole; FOX, cefoxitin; GEN, gentamicin; NAL, nalidixic acid; STR, streptomycin; SXT, trimethoprim/sulfamethoxazole; TET, tetracycline.

Supplementary Table S3. Antimicrobial resistance pattern in *Salmonella* Typhimurium (n = 553) isolated from livestock during 2019–2024 in South Korea

| Number of antimicrobials | % (No. of resistant isolates) | Most common pattern (No. of isolates) |
| --- | --- | --- |
| 0 | 46.3 (256) | – |
| 1 | 20.1 (111) | NAL (n = 88) |
| 2 | 9.6 (53) | AMP NAL (n = 37) |
| 3 | 3.6 (20) | AMP STR FIS (n = 7), |
|  |  | AMP CHL NAL (n = 7) |
| 4 | 3.1 (17) | AMP GEN FIS TET (n = 6), |
|  |  | AMP STR FIS TET (n = 6) |
| 5 | 4.7 (26) | AMP GEN NAL FIS TET (n = 6) |
| 6 | 5.1 (28) | AMP CHL STR FIS TET SXT (n = 11) |
| 7 | 4.2 (23) | AMP CHL NAL STR FIS TET SXT (n = 8) |
| 8 | 2.7 (15) | AMP CHL GEN NAL STR FIS TET SXT (n = 6) |
| 9 | 0.7 (4) | AMP CHL CIP GEN NAL STR FIS TET SXT (n = 3) |

AMP, ampicillin; CHL, chloramphenicol; CIP, ciprofloxacin; FIS, sulfisoxazole; GEN, gentamicin; NAL, nalidixic acid; STR, streptomycin; SXT, trimethoprim/sulfamethoxazole; TET, tetracycline.

Supplementary Table S4. Antimicrobial resistance pattern in *Salmonella* Enteritidis (n = 386) isolated from livestock during 2019–2024 in South Korea

| Number of antimicrobials | % (No. of resistant isolates) | Most common pattern (No. of isolates) |
| --- | --- | --- |
| 0 | 3.9 (15) | – |
| 1 | 37.3 (144) | NAL (n = 143) |
| 2 | 4.1 (16) | AMP NAL (n = 9) |
| 3 | 2.1 (8) | AMP NAL TET (n = 6) |
| 4 | 21.5 (83) | AMP NAL STR FIS (n = 73) |
| 5 | 18.4 (71) | AMP NAL STR FIS TET (n = 69) |
| 6 | 1.8 (7) | AMP CTX CAZ GEN NAL TET (n = 6) |
| 7 | 3.9 (15) | AMP FEP CTX CAZ GEN NAL TET (n = 14) |
| 8 | 0.8 (3) | AMP CTX CHL NAL STR FIS TET SXT (n = 2) |
| 9 | 6.2 (24) | AMP FEP CTX CAZ GEN NAL STR FIS TET (n = 19) |

AMP, ampicillin; CAZ, ceftazidime; CHL, chloramphenicol; CTX, cefotaxime; FEP, cefepime; FIS, sulfisoxazole; GEN, gentamicin; NAL, nalidixic acid; STR, streptomycin; SXT, trimethoprim/sulfamethoxazole; TET, tetracycline.

Supplementary Table S5. Antimicrobial resistance pattern in *Salmonella* Typhimurium variant (*S.* 1,4,[5],12:i:-) (n = 289) isolated from livestock during 2019–2024 in South Korea

| Number of antimicrobials | % (No. of resistant isolates) | Most common pattern (No. of isolates) |
| --- | --- | --- |
| 0 | 24.2 (70) | – |
| 1 | 10 (29) | FIS (n = 15) |
| 2 | 8.0 (23) | AMP NAL (n = 19) |
| 3 | 11.8 (34) | AMP STR FIS (n = 25) |
| 4 | 20.8 (60) | AMP STR FIS TET (n = 47) |
| 5 | 6.9 (20) | AMP CHL STR FIS TET (n = 7) |
| 6 | 9.0 (26) | AMP CHL STR FIS TET SXT (n = 15) |
| 7 | 4.8 (14) | AMP CHL NAL STR FIS TET SXT (n = 6) |
| 9 | 2.4 (7) | AMP CTX CHL GEN NAL STR FIS TET SXT (n = 5) |
| 10 | 1.7 (5) | AMC AMP CTX FOX CHL NAL STR FIS TET SXT (n = 5) |
| 11 | 0.3 (1) | AMP CTX FOX CHL CIP GEN NAL STR FIS TET SXT (n = 1) |

AMC, amoxicillin/clavulanic acid; AMP, ampicillin; CHL, chloramphenicol; CIP, ciprofloxacin; CTX, cefotaxime; FIS, sulfisoxazole; FOX, cefoxitin; GEN, gentamicin; NAL, nalidixic acid; STR, streptomycin; SXT, trimethoprim/sulfamethoxazole; TET, tetracycline.

Supplementary Table S6. Antimicrobial resistance pattern in *Salmonella* Agona (n = 268) isolated from livestock during 2019–2024 in South Korea

| Number of antimicrobials | % (No. of resistant isolates) | Most common pattern (No. of isolates) |
| --- | --- | --- |
| 0 | 14.6 (39) | – |
| 1 | 14.6 (39) | NAL (n = 19) |
| 2 | 3.0 (8) | CIP NAL (n = 4) |
| 3 | 1.1 (3) | AMP FIS TET (n = 1), |
|  |  | FIS TET SXT (n = 1), |
|  |  | FOX CIP NAL (n = 1) |
| 4 | 13.4 (36) | CHL STR FIS TET (n = 23) |
| 5 | 10.4 (28) | AMP STR FIS TET SXT (n = 19) |
| 6 | 41.0 (110) | AMP CHL STR FIS TET SXT (n = 104) |
| 7 | 0.4 (1) | AMP CTX CHL NAL STR FIS TET (n = 1) |
| 9 | 0.7 (2) | AMC AMP CTX CAZ CIP NAL STR FIS TET (n = 1), |
|  |  | AMP CTX CHL GEN NAL STR FIS TET SXT (n = 1) |
| 10 | 0.7 (2) | AMP CTX FOX CHL CIP NAL STR FIS TET SXT (n = 1), |
|  |  | AMP CTX FOX CHL GEN NAL STR FIS TET SXT (n = 1) |

AMC, amoxicillin/clavulanic acid; AMP, ampicillin; CAZ, ceftazidime; CHL, chloramphenicol; CIP, ciprofloxacin; CTX, cefotaxime; FIS, sulfisoxazole; FOX, cefoxitin; GEN, gentamicin; NAL, nalidixic acid; STR, streptomycin; SXT, trimethoprim/sulfamethoxazole; TET, tetracycline.

Supplementary Table S7. Antimicrobial resistance pattern in *Salmonella* Albany (n = 214) isolated from livestock during 2019–2024 in South Korea

| Number of antimicrobials | % (No. of resistant isolates) | Most common pattern (No. of isolates) |
| --- | --- | --- |
| 3 | 18.7 (40) | NAL FIS SXT (n = 40) |
| 4 | 7.9 (17) | NAL STR FIS SXT (n = 6) |
| 5 | 1.4 (3) | CIP NAL STR FIS SXT (n = 1), |
|  |  | CHL NAL STR FIS SXT (n = 1), |
|  |  | CHL CIP NAL FIS SXT (n = 1) |
| 6 | 52.8 (113) | AMP CHL NAL FIS TET SXT (n = 110) |
| 7 | 16.4 (35) | AMP CHL CIP NAL FIS TET SXT (n = 24) |
| 8 | 0.9 (2) | AMP CHL GEN NAL STR FIS TET SXT (n = 1), |
|  |  | AMP CHL CIP NAL STR FIS TET SXT (n = 1) |
| 9 | 0.9 (2) | AMP CTX CHL GEN NAL STR FIS TET SXT (n = 1), |
|  |  | AMP FOX CHL CIP NAL STR FIS TET SXT (n = 1) |
| 10 | 0.5 (1) | AMC AMP CTX FOX CAZ CHL NAL FIS TET SXT (n = 1) |
| 11 | 0.5 (1) | AMC AMP CTX FOX CAZ CHL NAL STR FIS TET SXT (n = 1) |

AMC, amoxicillin/clavulanic acid; AMP, ampicillin; CAZ, ceftazidime; CHL, chloramphenicol; CIP, ciprofloxacin; CTX, cefotaxime; FIS, sulfisoxazole; FOX, cefoxitin; GEN, gentamicin; NAL, nalidixic acid; STR, streptomycin; SXT, trimethoprim/sulfamethoxazole; TET, tetracycline.

Supplementary Table S8. Antimicrobial resistance pattern in *Salmonella* Montevideo (n = 195) isolated from livestock during 2019–2024 in South Korea

| Number of antimicrobials | % (No. of  resistant isolates) | Most common pattern (No. of isolates) |
| --- | --- | --- |
| 0 | 7.7 (15) | – |
| 1 | 72.8 (142) | NAL (n = 141) |
| 2 | 12.3 (24) | NAL FIS (n = 17) |
| 3 | 0.5 (1) | STR FIS TET (n = 1) |
| 4 | 2.6 (5) | GEN NAL STR FIS (n = 2) |
| 5 | 2.1 (4) | CIP NAL FIS TET SXT (n = 3) |
| 6 | 1.0 (2) | CIP NAL STR FIS TET SXT (n = 2) |
| 7 | 0.5 (1) | AMP FEP CTX CAZ GEN NAL TET (n = 1) |

AMP, ampicillin; CAZ, ceftazidime; CHL, chloramphenicol; CIP, ciprofloxacin; CTX, cefotaxime; FEP, cefepime; FIS, sulfisoxazole; GEN, gentamicin; NAL, nalidixic acid; STR, streptomycin; SXT, trimethoprim/sulfamethoxazole; TET, tetracycline.

Supplementary Table S9. Antimicrobial resistance pattern in *Salmonella* Senftenberg (n =132) isolated from livestock during 2019–2024 in South Korea

| Number of antimicrobials | % (No. of resistant isolates) | Most common pattern (No. of isolates) |
| --- | --- | --- |
| 0 | 11.4 (15) | – |
| 1 | 80.3 (106) | NAL (n = 106) |
| 2 | 0.8 (1) | NAL STR (n = 1) |
| 3 | 0.8 (1) | NAL STR FIS (n = 1) |
| 4 | 0.8 (1) | CHL STR FIS TET (n = 1) |
| 5 | 6.1 (8) | AMP CHL STR FIS TET (n = 6) |

AMP, ampicillin; CHL, chloramphenicol; FIS, sulfisoxazole; NAL, nalidixic acid; STR, streptomycin; TET, tetracycline.

Supplementary Table S10. Antimicrobial resistance pattern in *Salmonella* Bareilly (n = 114) isolated from livestock during 2019–2024 in South Korea

| Number of antimicrobials | % (No. of  resistant isolates) | Most common pattern (No. of isolates) |
| --- | --- | --- |
| 0 | 73.7 (84) | – |
| 1 | 20.2 (23) | FIS (n = 11) |
| 5 | 1.8 (2) | AMP CTX CHL NAL TET (n = 1), |
|  |  | AMP CHL STR FIS TET (n = 1) |
| 6 | 1.8 (2) | AMC AMP CTX FOX CAZ NAL (n = 2) |
| 8 | 0.9 (1) | AMP CHL GEN NAL STR FIS TET SXT (n = 1) |
| 9 | 1.8 (2) | AMP CTX CHL GEN NAL STR FIS TET SXT (n = 2) |

AMC, amoxicillin/clavulanic acid; AMP, ampicillin; CAZ, ceftazidime; CHL, chloramphenicol; CTX, cefotaxime; FIS, sulfisoxazole; FOX, cefoxitin; GEN, gentamicin; NAL, nalidixic acid; STR, streptomycin; SXT, trimethoprim/sulfamethoxazole; TET, tetracycline.

Supplementary Table S11. Antimicrobial resistance pattern in *Salmonella* Rissen (n = 92) isolated from livestock during 2019–2024 in South Korea

| Number of antimicrobials | % (No. of  resistant isolates) | Most common pattern (No. of isolates) |
| --- | --- | --- |
| 0 | 22.8 (21) | – |
| 1 | 20.7 (19) | NAL (n = 13) |
| 2 | 17.4 (16) | NAL FIS (n = 6) |
| 3 | 3.3 (3) | CHL NAL FIS (n = 2) |
| 4 | 8.7 (8) | AMP FIS TET SXT (n = 5) |
| 5 | 10.9 (10) | AMP CHL STR FIS TET (n = 5) |
| 6 | 10.9 (10) | AMP CHL CIP NAL STR TET (n = 2), |
|  |  | AMP CHL GEN NAL STR TET (n = 2), |
|  |  | GEN NAL STR FIS TET SXT (n = 2) |
| 7 | 1.1 (1) | AMP CHL GEN STR FIS TET SXT (n = 1) |
| 8 | 2.2 (2) | AMP CHL CIP NAL STR FIS TET SXT (n = 1), |
|  |  | AMC AMP CTX FOX CAZ GEN NAL STR (n = 1) |
| 9 | 2.2 (2) | AMC AMP CTX FOX CAZ GEN NAL STR FIS (n = 1), |
|  |  | AMP CTX CHL GEN NAL STR FIS TET SXT (n = 1) |

AMC, amoxicillin/clavulanic acid; AMP, ampicillin; CAZ, ceftazidime; CHL, chloramphenicol; CIP, ciprofloxacin; CTX, cefotaxime; FIS, sulfisoxazole; FOX, cefoxitin; GEN, gentamicin; NAL, nalidixic acid; STR, streptomycin; SXT, trimethoprim/sulfamethoxazole; TET, tetracycline.
